# Report on Exemptions in Oneida County

**Published:** 1863-01

**Authors:** Ira D. Hopkins

**Affiliations:** Examining Surgeon’s Office for Oneida County, Utica


### REPORT ON EXEMPTIONS IN ONEIDA COUNTY.

To the Editor of the Buffalo Medical and Snrgical Journal:

You are at liberty to publish in your columns the following report of my examinations. The report is nearly a fac simile of the one I sent to the Surgeon-General:

*Examining Surgeon's Office for Oneida County,*
Utica, December 9th, 1862.

S. Oakley Vanderpoel, M. D., Surgeon-General, Albany:

Sir:—In obedience to the official instructions accompanying my commission, I have the honor of transmitting to you the subjoined report.

I entered upon the discharge of the duties of the position assigned to me on the 20th day of October, that being as soon as the Commissioner was ready, and continued my examination until the 22d day of November.

By order from the Adjutant General, I have examined some seven hundred affidavits taken by the Commissioner to determine whether or not the complaints stated in said affidavits are sufficient cause for exemption. In six hundred and seventeen of the said affidavits the complaints stated therein, if true, are, in my opinion, sufficient to entitle the applicants to exemption.

In consequence of the large and disorderly crowd awaiting an entrance to my office, I was obliged to secure the services of a police officer. The Chief of the Police detailed three subordinates to prevent the blockrding of the street and to secure to the merchants and pedestrians their accustomed privileges unmolested. In compliance with the earnest solicitations of the merchants in the immediate vicinity, his honor the Mayor, ordered me to remove my examining office to the Court House.

Dr. Luther Guiteau having declined to serve, and no other appointment having been made, my labor thereby was considerably more arduous and tedious than it otherwise would have been, and in order to do justice to those wishing examinations, and to complete my mission in the limited time alloted me, I was obliged to examine twelve hours, instead of seven hours a day, as I advertised.

Four thousand and twenty-seven applicants were examined, of which nine hundred and eighty-six were recommended to the Commissioner for exemption, and three thousand and forty-one were rejected. The ratio of exemption is 986–4.027.

*Ages.*—Between the age of 18 and 20, 40; between 20 and 25, 105; between 25 and 30, 183; between 30 and 35, 210; between 35 and 40, 252; between 40 and 45, 190; between 45 and 50, 6.

*Nativity.*—American, 727; Welch, 73; English, 59; German, 55; Irish, 54; French, 10; Scotch, 8.

*Occupations.*—Agents, 8; artists, 2; book-keepers, 9; blacksmiths, 18; bankers, 3; boot and shoe makers, 25; bakers, 4; book binders, 1; butchers, 17; builders, 9; carpenters, 33; clerks, 59; carriage makers, 9; constables, 2; conductors, 3; cigar makers, 9; cabinet makers, 3; coopers, 3; dentists, 5; editors, 2; engineers, 3; engravers, 1; farmers, 410; hotel keepers, 8; harness makers, 8; lawyers, 16; laborers, 57; livery keepers, 2; moulders, 20; merchants, 97; machinists, 29; mechanics, 11; masons, 5; millwrights, 2; manufacturers, 2: millers, 6; oil refiners, 1; photographers, 3; physicians, 13; painters, 9; pedlers, 5; printers, 8; pattern makers, 4; spinners, 7; superintendents, 4; students, 10; saloon keepers, 4; tailors, 12; tinsmiths, 4; teachers, 3; no trade, 8.

Below I give a plain and general report, rather than a scientific classification, of the diseases upon which recommendation for exemption was made:

| | Surgeon's. | Com'rs. | Refused. |
|---|---|---|---|
| Asthma | 26 | 7 | 23 |
| Disease of bowels, | 42 | 6 | 48 |
| Disease of throat and lungs, | 178 | 71 | 249 |
| Disease of heart, | 66 | 24 | 90 |
| Disease of kidneys and bladder, | 34 | 15 | 49 |
| Disease of testes, | 4 | 4 | 8 |
| Defective hearing, | 78 | 31 | 109 |
| Defective speech, | 7 | 5 | 12 |
| Defective vision and loss of sight, | 82 | 40 | 122 |
| Dementia, | 4 | -- | 4 |
| Debility, general ill health, | 34 | 14 | 48 |
| Fistula in ano, | 6 | -- | 6 |
| Epilepsy, | 22 | 11 | 33 |
| Hemorrhoids and disease of rectum, | 42 | 40 | 82 |
| Hernia, | 87 | 73 | 160 |
| Injury to joints, | 69 | 67 | 136 |
| Injury to limbs, | 26 | 66 | 92 |
| Injury to hands and feet, | 34 | 37 | 71 |
| Lumbar abscess, | 3 | -- | 3 |
| Paralysis, | 3 | -- | 3 |
| Rheumatism, | 42 | 45 | 87 |
| Sciatica, | 1 | -- | 1 |
| Spinal affection, | 24 | 14 | 38 |
| Syphilis, | 20 | 12 | 32 |
| Varicose veins, | 22 | 12 | 34 |
| Varicocele, | 14 | 12 | 26 |
| All other causes, | 16 | 11 | 27 |
| Total, | 986 | 617 | 1603 |

*Height.*—Five feet 6 inches and under 6 feet, 716; under 5 feet 6 inches, 198; 6 feet and over, 77.

*Weight.*—Between 90 and 100 pounds, 3; between 100 and 110, 12; between 110 and 120, 12; between 120 and 130, 158; between 130 and 140, 220; between 140 and 150, 190; between 150 and 160, 160; between 160 and 170, 113; between 170 and 180, 52; between 180 and 190, 30; between 190 and 200, 18; between 200 and 210, 10; between 220 and 230, 4; between 230 and 240, 4.

*Color of Eyes.*—Blue, 366; grey, 338; black, 158; brown, 90; hazel, 34.

*Color of Hair.*—Brown, 742; black, 190; grey, 22; red, 32.

*Complexion.*—Fair, 616; dark, 370.

My examinations having been thorough and rigid, exercising as I did my best medical ability, and relying almost entirely upon my own judgment, except in cases of obscure disease, not apparent upon a single examination, it is probable that some were rejected during the pressure whose claims for exemption were just, and who ought to have been recommended.

I endeavored to follow your instructions and discharge the duties of the office faithfully.

I am, very respectfully yours,

IRA D. HOPKINS, JR.

